# Mechanical Alignment in Total Knee Arthroplasty for Varus Knee Osteoarthritis Leads to Significant Tibial Bone Loss

**DOI:** 10.7759/cureus.30107

**Published:** 2022-10-09

**Authors:** Ashim Mannan, Ahmed Y Saber, Ben Waterson, Andrew Roberton, Andrew Toms

**Affiliations:** 1 Exeter Knee Reconstruction Unit - Princess Elizabeth Orthopaedic Centre, Royal Devon and Exeter NHS Foundation Trust, Exeter, GBR

**Keywords:** anatomical alignment, mechanical alignment, total knee arthroplasty technique, varus knee, tibial cut, knee arthroplasty

## Abstract

Background

Obtaining a neutral postoperative alignment is said to be a guiding principle for performing a successful total knee arthroplasty (TKA). There are many different alignment philosophies and surgical techniques to attain the goal of proper alignment. This study aimed to radiologically measure the difference in the amount of tibial bony resection required to perform a mechanical alignment versus an anatomic alignment TKA.

Methods

Two observers retrospectively reviewed the long leg radiographs of 100 patients (61 females and 39 males) listed for TKA between 2015 and 2018, measuring the amount of tibial bony resection required to achieve mechanical or anatomic alignment TKA.

Results

These radiographs' overall lower limb mechanical axis ranged between 16° varus and 17.6° valgus (mean 4.4° varus, standard deviation (SD) 6.64). By referencing 4 mm from the worn side, the mean resection needed from the normal side of the tibial plateau is 7.6 mm in the mechanical alignment measurement and 5.2 mm in the anatomical alignment measurement (p<0.0001). Therefore, 17% of mechanical alignment cuts require a tibial cut of more than 10 mm (mean 12.382 mm). No anatomical alignment measurements exceed 10 mm. When a virtual tibial cut >10 mm is required, the medial proximal tibial angle (MPTA) is a stronger predictor of deformity than the mechanical axis.

Conclusion

This radiological study shows that an anatomical alignment tibial cut is more bone conserving on the tibia than a mechanical alignment tibial cut and may lead to less asymmetry of the bony cuts and greater bony preservation, but clinical correlation is needed.

## Introduction

Traditionally, the neutral mechanical axis has remained the established alignment target in total knee arthroplasty. Historical literature misleadingly suggested higher rates of survival in total knee arthroplasties (TKAs) that fell within a post-operative hip-knee-ankle (HKA) angle +/- 3 degrees in the coronal plane [1].

The concept of “constitutional varus” has recently become more widely accepted [2]. This refers to the pre-arthritic anatomy of a proportion of arthritic knees that are in varus alignment rather than neutral or valgus alignment and is seen to be around 32% in men and 17% in women [3].

A significant number of patients undergoing total knee replacement have primary tibia vara. Furthermore, more recent evidence suggests superior clinical outcomes in knees left in slight varus compared to a true mechanical alignment [4]. We need to consider the implications of a truly mechanical axis cut in the tibia in this population of patients.

Kinematic knee arthroplasty was first described in 2006 and aimed to restore the native or constitutional alignment of the knee [4]. Further analysis of this concept is warranted, as well as that of joint obliquity. Patients with a constitutional varus corrected to a neutral mechanical axis experience a strain more significant than that found within the native knee [5]. The tibial component is aligned with the flexion, rotational and varus-valgus axes with reference to the proximal tibial articular surface [6].

The concept of anatomic alignment (AA) was first described and expanded upon by Hungerford and Krackow [7]. The aim of AA TKA is to recreate the natural obliquity of the anatomic joint line in relation to the mechanical axis of the lower extremity [7]. The tibial resection plane conforms to the native joint line, allowing for improved load distribution and preservation of native joint line obliquity [8]. This technique allows for the optimisation of patellofemoral biomechanics with balancing lateral retinacular ligament tension throughout knee flexion [9].

For this paper, we will use the term ‘anatomic alignment’ when referring to knees left in post-operative varus rather than the term ‘kinematic alignment’. Anatomic alignment is a concept that can be measured using plain full-leg length radiographs, whereas kinematic alignment is a surgical principle using staged resection during the operation and considers the articular cartilage [10].

The true mechanical axis in TKA conforms to the HKA angle of zero degrees, with the femoral cut perpendicular to the femoral mechanical axis (FMA) and the tibial cut perpendicular to the tibial mechanical axis (TMA) [10]. Usually, a ‘standard’ femoral valgus cut of 5 or 6 degrees and a ‘classic tibial cut’ according to extramedullary referencing is completed [11]. In most patients, completing femoral and tibial cuts according to true mechanical principles changes the obliquity and level of the joint line [8]. To minimise ligament imbalance, a compensatory adjustment in the femoral axial rotation is required. However, placing the femoral component away from the joint line causes suboptimal patellofemoral and tibiofemoral kinematics with resultant pain, instability and stiffness [9].

The ‘classic’ tibial resection in the coronal plane follows a 4 mm/9 mm cut according to the worn and preserved tibial articular surface although this can be modified [11]. The majority of pre- and post-operative assessment is still determined by the short limb radiograph of the knee, and therefore, verification of a neutral tibial mechanical axis in surgical planning is limited to research trials and the relatively finite number of centres that include full-length limb radiographs as part of peri-operative criteria and imaging [12]. Surgeons who subscribe to a mechanical alignment aim but use a routine standard 4 mm/9 mm cut in a patient with primary tibial genu varus will not achieve mechanical alignment of the tibia; however, it is not clear in the literature what the result of a truly mechanical axis (MA) tibial cut would be.

Therefore, this study aimed to accurately measure the degree of bony resection required to achieve a true neutral tibial mechanical cut from a series of varus arthritic knee radiographs. It is compared to a tibial anatomic resection plane using the guiding principle of preservation of the joint line.

## Materials and methods

Between 2015 and 2018, 446 patients listed for TKA received long-leg radiographs as part of the routine pre-operative assessment. Exclusion criteria included patients with complex knee arthritis or deformity requiring stemmed or hinge implants and patients younger than 50. The radiographs were performed at a single institution.

All patients underwent full-length digital radiography with subjects standing barefoot with the patellae orientated forward. Three consecutive ankle, knee and hip images were acquired from a distance of 260 cm with a 35-squared detector plate. All radiographic images were digitally acquired using a picture archiving and communications system (PACS). Radiographic measurements were completed using PACS software with a minimum detectable angle of 0.1 degrees and a distance of 0.1 mm.

**Figure 1:**
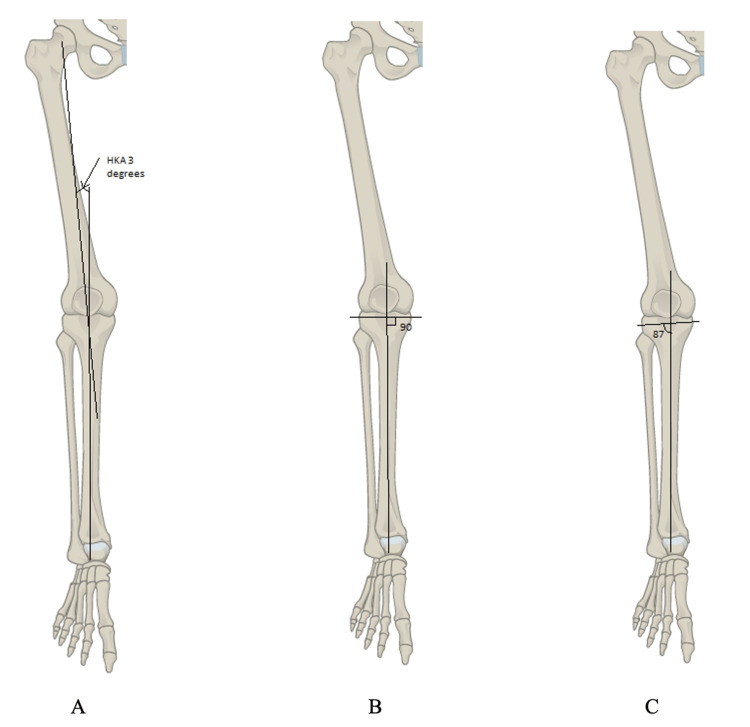
A: The hip-knee-ankle angle; B: The angle in the mechanical cut; C: The angle in the anatomical cut

**Figure 2:**
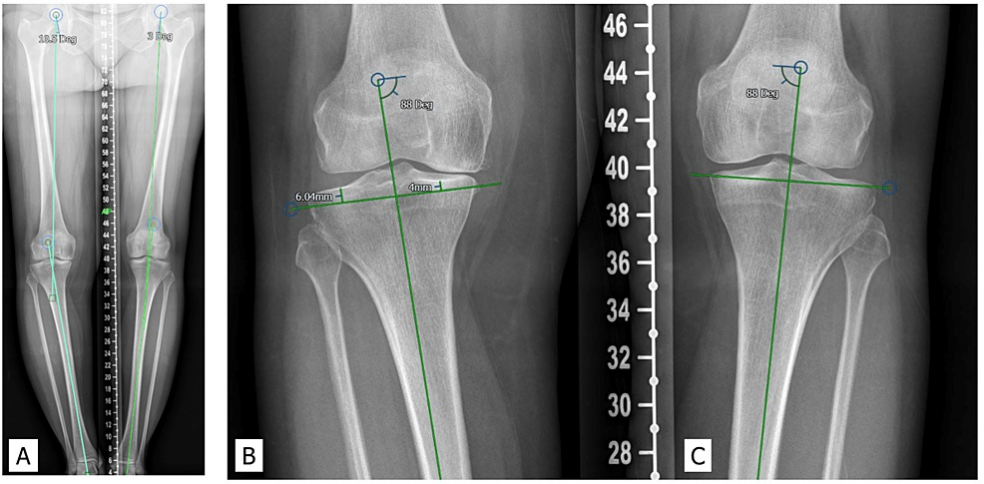
A: X-ray shows how to measure the hip-knee-ankle angle; B and C: Anatomical resection for a patient

**Figure 3:**
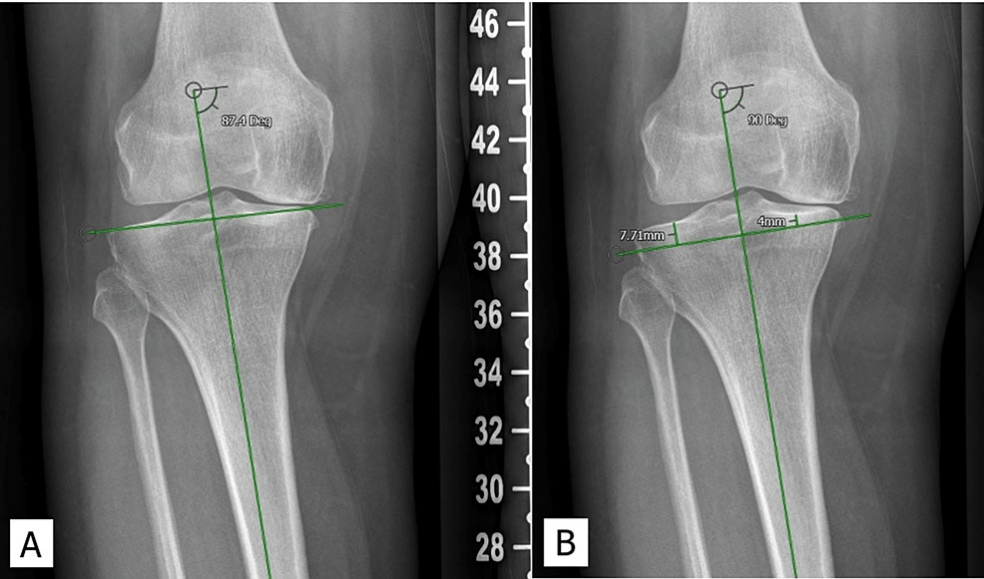
A: The measurement of MTPA; B: Mechanical resection for the same patient shown in Figure 2 MTPA: medial proximal tibial angle

In total, six radiographic parameters were measured in each included case. The overall HKA of the affected and contralateral limbs, the medial proximal tibial angle (MPTA) of the affected and unaffected knee, and the measurement in mm of required resection when referencing a resection of the worn side of 4 mm using both mechanical and anatomic principles (Figures 1-3).

The virtual resection plane was aligned at right angles to the tibial mechanical angle (TMA) with the mechanical tibial cut. The virtual anatomic cut resection plane referenced the contralateral MPTA. Therefore, only patients with unilateral radiographic arthritis deformity were included in this study. 

Although established principles of anatomic knee arthroplasty involve planning and templating with a pre-operative MRI or CT and estimation of femoral bony cuts according to pre-arthritic geometry and then proceeding with pre-determined bony cuts according to either patient-specific moulds, calliper-based measurements and now robotics, the uptake of this technology has been limited due to its complexity and potential lack of reproducibility. The authors, therefore, adopted a modified tibial template, which considers the weight-bearing axis of the knee and commits to principles of preservation of joint line obliquity and articular surface planes.

## Results

From 446 radiographs, 100 patients (61 females and 39 males) satisfied the inclusion criteria of radiographic arthritis to enable reference to the contralateral pre-arthritic joint. The mean age was 68.7 years (range 50 - 84, SD 8.7). HKA ranged between 16 varus and 17.6 valgus (mean 4.4 varus, SD 6.64). Comparisons between male and female ipsilateral HKA confirmed male subjects to be significantly more varus (Table 1).

**Table 1:** HKA values males vs females (unpaired T-test p = 0.0414: males significantly more varus) HKA: hip-knee-ankle

	Mean	SD
Male HKA	6.1	5.25
Female HKA	3.3	7.22

Values for ipsilateral and contralateral MPTA are outlined in Table 2. A significant correlation was demonstrated to be present between the arthritic limb HKA and MPTA using Pearson’s correlation coefficient (r =-0.6581 P-Value < 0.00001) (Table 2).

**Table 2:** Values for ipsilateral and contralateral MPTA MPTA: medial proximal tibial angle

	Mean	Range	SD
Ipsilateral MPTA	86.6	74.9 – 94.9	3.11
Contralateral MPTA	87.6	80.5 – 93.6	2.35

Further sub-group analysis of the arthritic limb demonstrated 23 patients with HKA of 0-3 degrees varus and 66 patients with an HKA > 3 degrees varus. Subgroup correlation of HKA vs MPTA within these groups using Pearson’s correlation test showed a lack of significant correlation in the HKA 0 - 3 group (R = 0.1806, p = 0.409576) and a highly significant correlation in the HKA > 3 group (R = -0.5594, P < 0.00001). The correlation, therefore, between HKA and MPTA in the arthritic limb is demonstrated to be affected by more significant deformities.

Comparing mechanical and anatomic tibial cuts, while referencing 4 mm of resection from the worn side, the mean height of necessary resection from the unworn side was 7.6 mm in the mechanically aligned and 5.2 mm in the anatomically aligned group. Unpaired t-test comparison demonstrated this difference to be significant (p < 0.0001). In total, 46% of patients required a > 7.5 mm cut in the mechanical group and 16% in the anatomic group (Table 3).

**Table 3:** Values for mechanical and anatomic resection planes

Alignment	Mean	Range (mm)	SD	SEM	N
Anatomic cut	5.21	0 – 9.66	2.18	0.22	100
Mechanical cut	7.58	0 – 28.20	3.33	0.33	100

**Figure 4:**
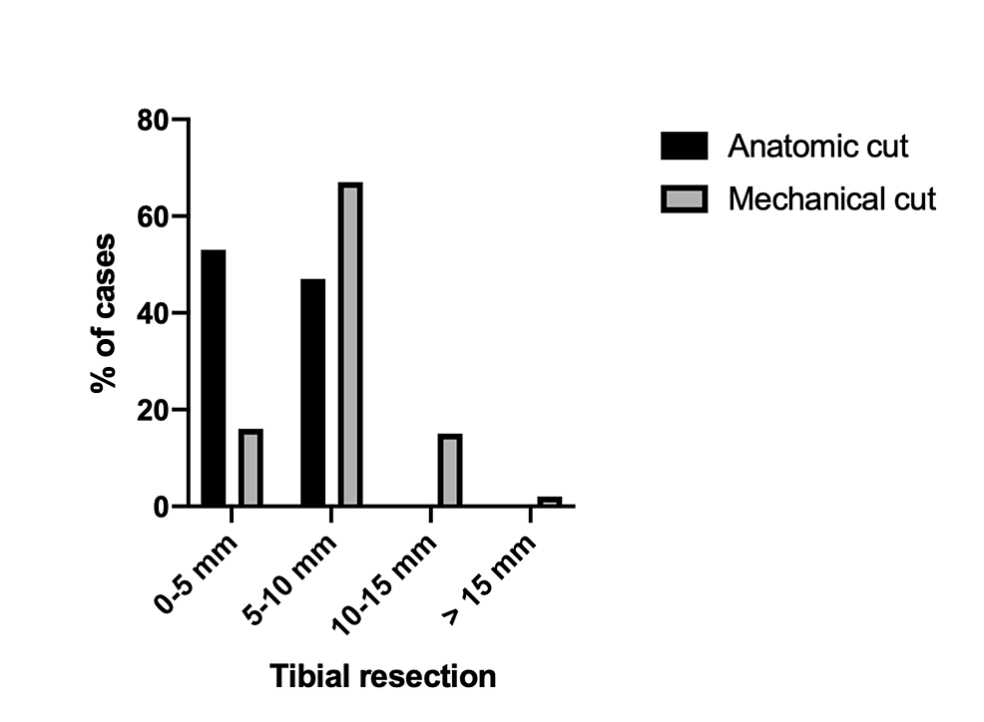
Range values of required tibial resection: mechanically aligned versus anatomic groups

Range values of necessary resection between anatomic and mechanically aligned cuts are outlined in Table 4. Seventeen per cent (17%) of cases within the mechanically aligned group required a tibial cut > 10 mm, whereas no patients within the anatomic group fell in this category (Table 4, Figure 4).

**Table 4:** Range values of required resection mechanically aligned vs anatomic groups

Alignment	0-5mm	5-10mm	10-15mm	> 15mm
Anatomic cut	53%	47%		
Mechanical cut	16%	67%	15%	2%

Subgroup analysis of cases requiring a mechanical cut of >10 mm

As outlined, with anatomic resection parameters, no patients required a virtual resection cut of > 10 mm; however, within the mechanical alignment group, 17 cases required increased resection planes (mean 12.382 mm). The table below outlines data specific to the two groups with statistical comparisons using the student's t-test (Table 5).

**Table 5:** Data values extracted from the two groups: mechanical cut height of either >/< 10 mm *student's t-test (significant values in bold)

	Mechanical cut > 10mm	Mechanical cut < 10mm	P *
Total	17	83	
Mean	12.382	6.593	< 0.00001
Mean ipsilateral HKA	6.7535	3.8964	0.053
Mean contralateral HKA	3.2588	2.9024	0.29
Mean ipsilateral MPTA	84.482	87.8831	0.008
Mean contralateral MPTA	85.953	87.8831	0.00087
Mean Anatomic cut	7.0412	4.8324	0.000043

Table 5 demonstrates that while using mechanical alignment principles and when larger tibial cuts (>10 mm) are required, a closer association is seen with the MPTA (vs HKA), therefore affirming the “intra-articular tibia” as the primary “driver” of deformity within this group. Additionally, within the (>10 mm) tibial resection group from all cases, the overall mean tibial cut while applying mechanical vs anatomic principles is significantly greater (12.38 mm vs 7.04 mm).

Using Pearson’s correlation coefficient applied to all cases within the dataset, the table below describes correlations between HKA and MPTA (both ipsilateral and contralateral) with the final virtual anatomic or mechanical resection cut.

Table 6 shows a significant correlation between the ipsilateral MPTA with regard to the virtual anatomic and mechanical cut size. In addition, the HKA correlates significantly with the final virtual mechanical cut. 

**Table 6:** Data values correlation to anatomic vs mechanical resection (Pearson’s correlation coefficient, significant values in bold)

	Anatomic cut	Mechanical cut
Ipsilateral HKA	R = 0.1136 (p=0.26)	R = 0.3302 (p=0.000793)
Contralateral HKA	R = 0.008 (p=0.43)	R = 0.17 (p=0.09)
Ipsilateral MPTA	R=-0.209 (p=0.0369)	R= -0.612 (p<0.00001)
Contralateral MPTA	R= 0.0557 (p=0.582)	-0.5411 (p<0.00001)

## Discussion

Does mechanical alignment lead to excessive resection of bone in varus knees? Yes, in 17% of cases, the tibial cut resects greater than 10 mm of the tibial bone.

The authors demonstrate that completing a true neutral tibial mechanical resection cut in TKA leads to wide bony gaps, asymmetric bony resection and non-constitutional joint line. This subsequently leads to an imbalance in collateral ligaments that sometimes necessitates extensive release and changes the joint obliquity [13]. The lateral tibial plateau resection range is sometimes extensive, with 46% of cases requiring a > 7.5 mm lateral tibial plateau resection plane.

Kinematic alignment in knee arthroplasty is a concept that aims to align the single-radius femoral component with the three-dimensional axis of the knee [14]. This differs from the mechanical axis of the knee; the common goal is to achieve a well-balanced knee by performing bony cuts alone. Deviations from neutral resection planes should exist within broadly accepted parameters. In contrast, the mechanical alignment concept is referenced solely to the coronal axis. The coronal axis is unlikely to be the primary determinant of success in three-dimensional anatomy.

Kinematic knee arthroplasty, popularised by Howell et al., relies on either pre-operative MRI imaging that provides a three-dimensional template for bony resection planes [15], or staged manual resection planes that are measured with callipers [16] or a functional technique using ligament tension. The former method relies on patient-specific “rapid prototyping” technology [17]. Despite numerous reported data-synthesis and analyses of this technology, PSI has not proven to achieve sufficient reproducible accuracy in knee arthroplasty [18]. Additionally, the exact calliper method involves a steep learning curve and is prone to error due to its multiple dependent steps.

The authors propose that referencing from weight-bearing imaging offers optimum templating. Weight-bearing long limb radiographs have been shown to confer useful utility and accuracy when standardised techniques are used such as in our study. Babazadeh et al. demonstrated long-limb radiographs to be a reliable imaging modality when compared with CT with excellent intra and inter-observer correlation [19].

Interestingly, they found computer navigation data registration to be less reproducible. Good agreement between CT scanograms and CT scout scans has been demonstrated; however, they confirmed an overall under-diagnosis of malalignment in supine CT imaging [20]. Holme TJ et al. demonstrated inaccuracies in the measurement of the anatomical and mechanical axes of the limb with scout CT when compared to WB radiographs [21]. The methodology of this study highlights the importance of planning or templating with the weight-bearing alignment of the lower limb.

Historical papers asserted that with knee arthroplasty, malalignment outside a neutral mechanical axis results in increased shear stress upon polyethylene components leading to premature wear and aseptic loosening [22]. However, using more modern implants and polyethylene implant survivorship at 15 years has been shown not to differ significantly between neutral alignment and varus/valgus outliers [23,24].

Patient-reported satisfaction outcomes from neutral alignment studies remain suboptimal, with 33-54% of patients describing pain or residual symptoms [25]. Computer navigation improved the accuracy and ability to achieve MA, but this was never translated into improved patient outcomes. A meta-analysis done on a limited number of studies using kinematic knee arthroplasty compared with conventional alignment techniques has demonstrated some improved early results (distance walked before discharge) [26].

Overall, the HKA angle in the arthritic limb was shown to be a mean of 6.1 vs 3.3 degrees in males versus females (significantly greater in males). Bellemans et al. also demonstrated the varus angle to be greater in the male pre-arthritic knee [3]. The MPTA was shown to be our study's ‘driver’ of varus deformity in the arthritic knee. Bellemans demonstrated the MPTA and mechanical lateral distal femoral angle (mLDFA) to be the main predictors of constitutional varus in their study (40.8 and 29.4%, respectively) [3].

From our study, 17% of all cases required a tibial resection depth of > 10 mm using the mechanical concept, whereas the maximum resection required with the anatomic technique was 9.66 mm. Within this group, the mean ipsilateral HKA was 6.75 and MPTA 84.4. Again, the MPTA appeared to be the ‘driver’ of severe varus deformity within this group; the mean required resection depth being 12.3mm. The authors propose that it is within this group of severe varus deformities that mechanical alignment principles may lead to alteration of the joint line, significant bone loss and ligamentous instability.

Alignment strategies in TKA are subject to differing nomenclature. Examples of established methods include ‘mechanical alignment, anatomical alignment, adjusted mechanical alignment, kinematic alignment, restricted kinematic alignment and individualised alignment’. The method described in this study would conform with ‘anatomic alignment’. However, varying nomenclature to describe various techniques creates confusion and standardisation is required.

The limitations of this study include the lack of inter-observer verification of measured values. A further limitation is the use of X-ray imaging, which, despite excellent validation and accuracy, may be influenced by the rotational position of the lower limb. However, a standardised and validated technique of positioning and image capture was used in this study, with the same technique applied to all cases.

## Conclusions

Aiming to align the knee to the mechanical axis in patients with significant tibial varus results in a tibial bony resection of greater than 10 mm in 17% of our study population, whereas no patients within the anatomic group fell in this category. In practice, this will necessitate a large medial release to balance the prosthesis. Modern literature does not support the assertion that deviation from MA results in the early failure of the prosthesis. According to anatomic principles, the tibial resection plane leads to less asymmetry of the bony cut and greater bony preservation. Optimum alignment in TKA is an evolving area of interest in knee arthroplasty surgery. With the increasing use of robotic surgery, it is becoming possible to plan and deliver an alignment individualised to each patient more accurately. It offers the opportunity to preserve bone and potentially re-creates alignment more closely related to the joint in the pre-arthritic state. Continuing research is required to conclude clinical outcomes and survivorship within the ‘anatomically aligned’ total knee.
